# Dynamics of *Salmonella* Dublin infection and antimicrobial resistance in a dairy herd endemic to salmonellosis

**DOI:** 10.1371/journal.pone.0318007

**Published:** 2025-01-23

**Authors:** Victor Santos do Amarante, Joelma Kellen de Castro Pereira, Matheus Ferreira Serafini, Carolina Pantuzza Ramos, Isabela Pádua Zanon, Thayanne Gabryelle Viana de Souza, Tiago Facury Moreira, Antônio Ultimo de Carvalho, Rodrigo Melo Meneses, Flavia Figueira Aburjaile, Vasco Azevedo, Elias Jorge Facury Filho, Rodrigo Otávio Silveira Silva

**Affiliations:** 1 Veterinary School, Federal University of Minas Gerais, Belo Horizonte, Minas Gerais, Brazil; 2 Instituto de Ciências Biológicas, Federal University of Minas Gerais, Belo Horizonte, Minas Gerais, Brazil; University of Illinois Urbana-Champaign College of Veterinary Medicine, UNITED STATES OF AMERICA

## Abstract

*Salmonella* Dublin is a serovar that causes severe infections and cattle. Despite the importance of this agent, research on achieving its elimination from dairy farms is limited, which complicates risk mitigation and control efforts. This study thus aimed to assess the prevalence of *S*. Dublin on a farm with a history of outbreaks, to understand the dynamics of the infection, characterize the antimicrobial resistance of the isolates, and evaluate their genetic similarity. Multiparous cows in the postpartum phase are nearly five times more likely to shed *Salmonella* sp. A total of 39 cases of fatal septicemic salmonellosis caused by *S*. Dublin were confirmed in calves aged 3–5 months. Antimicrobial susceptibility was evaluated in 45 strains of *S*. Dublin, with 48.9% of the isolates classified as multidrug resistant, including resistance to penicillin (48.9%), tetracyclines (42.2%), and fluoroquinolones (33.3%). Seven multidrug-resistant isolates were selected for genomic sequencing. Among the resistance determinants identified, a mutation in the *gyrA* gene, present in all sequenced isolates, was notable. Analyses of cgMLST and SNPs revealed that the isolates from healthy animals were closely related to those found in animals with confirmed cases of *S*. Dublin, confirming that the agent was circulating among healthy animals across various categories. A high similarity was also found between the isolates in this study and strains causing salmonellosis in humans in Brazil, thus reinforcing the zoonotic nature and possible epidemiological link between cattle, and the occurrence of this disease in humans.

## Introduction

Among the more than 2,500 serovars of *Salmonella*, *Salmonella* Dublin stands out for its association with enteric, respiratory, septicemic, and reproductive disorders in cattle, resulting considerable economic losses [1–3]. Salmanellosis affects herds worldwide, while its control is complicated by the adaptation of *S*. Dublin to the bovine host, including the presence of chronic carriers that shed the agent into the environment through the feces, milk, and colostrum [4–7].

In addition to its importance in cattle, *S*. Dublin is a leading cause of severe invasive salmonellosis in humans, with a high mortality rate among in patients with underlying chronic diseases [8–11]. Recent studies in Brazil have indicated a strong molecular similarity between *S*. Dublin isolates from animals and those from infected humans, reinforcing the hypothesized epidemiological link between salmonellosis in cattle and humans [12, 13].

The increasing antimicrobial resistance of *Salmonella* spp. isolates has heightened the challenge of treating both animals and humans [5, 11, 14]. Studies have indicated an increase in multidrug-resistant *S*. Dublin isolates with decreased susceptibility to quinolones and third-generation cephalosporins, which are important antimicrobial classes for the treatment of severe *Salmonella* spp. infections in humans [10].

Despite the importance of *S*. Dublin in cattle and as a cause of infection in humans, studies assessing the prevalence and dynamics of this serovar in herds remain scarce [12, 15–17]. Further, little is known about the epidemiology of *S*. Dublin in carrier cattle, particularly among herds with clinical cases of the disease. Thus, the present study aimed to evaluate the prevalence of *S*. Dublin on a farm with a history of salmonellosis, to understand the infection dynamics, characterize the antimicrobial resistance of the isolates, and assess their genetic similarity.

## Material and methods

### Farm and animals

This study was conducted using samples collected on a dairy farm located in the central-west region of Brazil that ranked among the top ten milk producers in the country. The herd comprised Holstein cattle, with an average daily milk production of 29,000 liters, collected from approximately 900 lactating animals, each milked three times a day. Calves were housed in raised cages with slotted floors and fed discarded milk. During the rearing phase, the animals were moved to paddocks in a non-rotational system. Adult animals remained in a free-stall confinement system.

The farm in question was chosen for the study because it had reported cases of *S*. Dublin in the six months preceding the study. The cases were concentrated in animals aged 91 and 150 days and were characterized by lethargy, respiratory distress, jaundice, fever, and nearly 100% mortality. Upon necropsy, the affected animals commonly showed hepatomegaly, splenomegaly, and marked pulmonary congestion, as well as signs of sepsis. The diagnosis was repeatedly confirmed by isolating the bacteria from the extraintestinal organs of the affected animals and serotyping [18]. This study was approved by the Ethics Committee on Animal Use of the Federal University of Minas Gerais (protocol 176/2019). All procedures were carried out following the signing of an Informed Consent Form.

### Prevalence of *Salmonella* spp. in dairy herds (S1)

**Fig 1:**
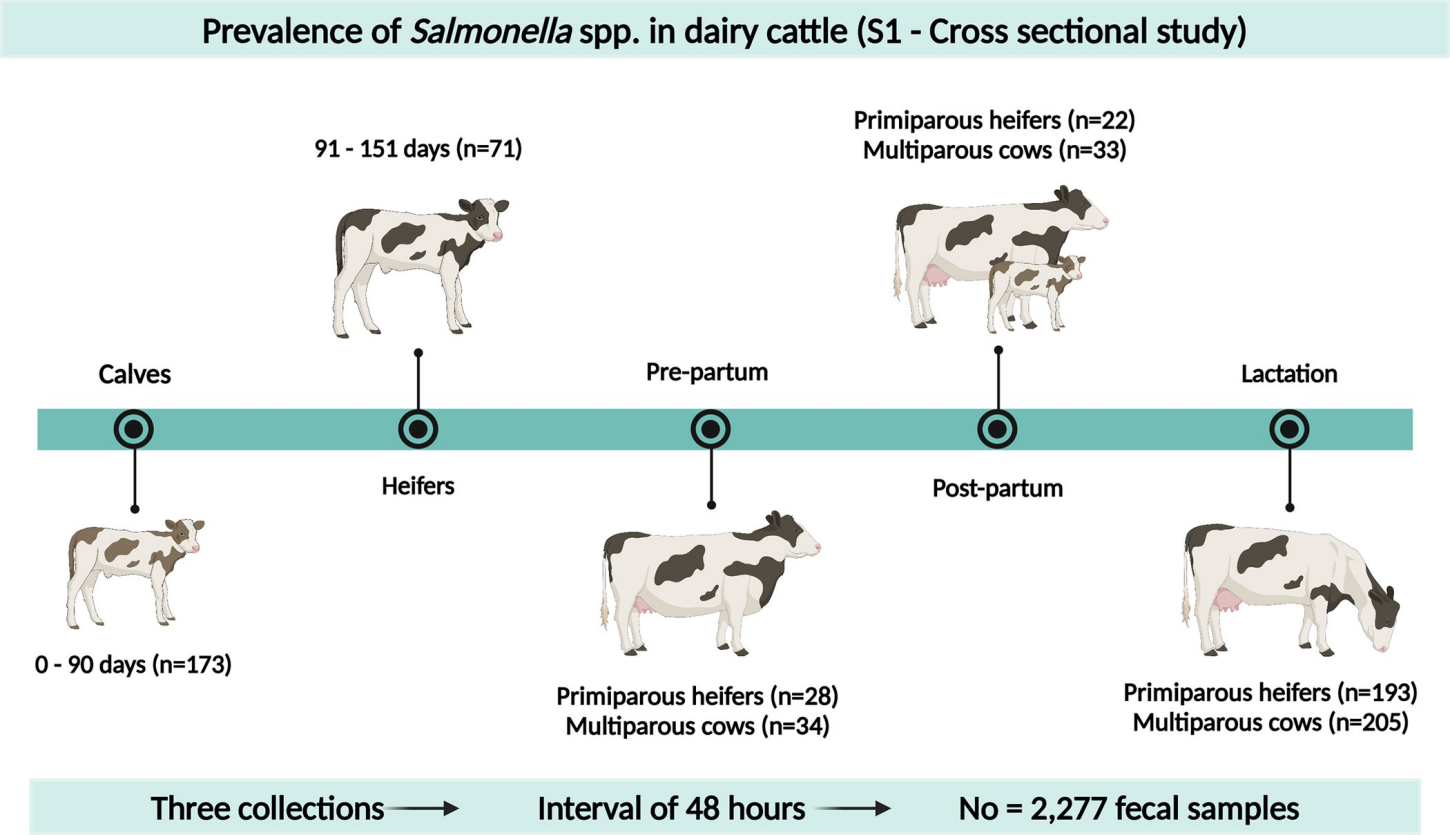
Graphical representation of the fecal sampling scheme in all of the animal categories evaluated, including the numbers of animals sampled in the cross-sectional study (S1).

To compare the shedding of *S*. Dublin across different farm categories, a cross-sectional study was conducted using samples from various categories. The sample size was determined based on the population size of the farm, an estimated prevalence rate of 5%, and a 95% confidence interval [19]. Samples were collected from the following categories: calves (0–90 days, n = 173), rearing (91–150 days, n = 71), prepartum (animals ≤30 days before the expected calving date; primiparous, n = 28; and multiparous, n = 34), postpartum (animals 1–30 days postpartum; primiparous, n = 22; and multiparous, n = 33), and lactation (primiparous, n = 193; and multiparous, n = 205). Each animal was sampled three times, at 48-hour intervals, yielding a total of 2,277 samples from 759 animals (Fig 1).

### Prevalence of *Salmonella* spp. in cows in the peripartum period (S2)

**Fig 2:**
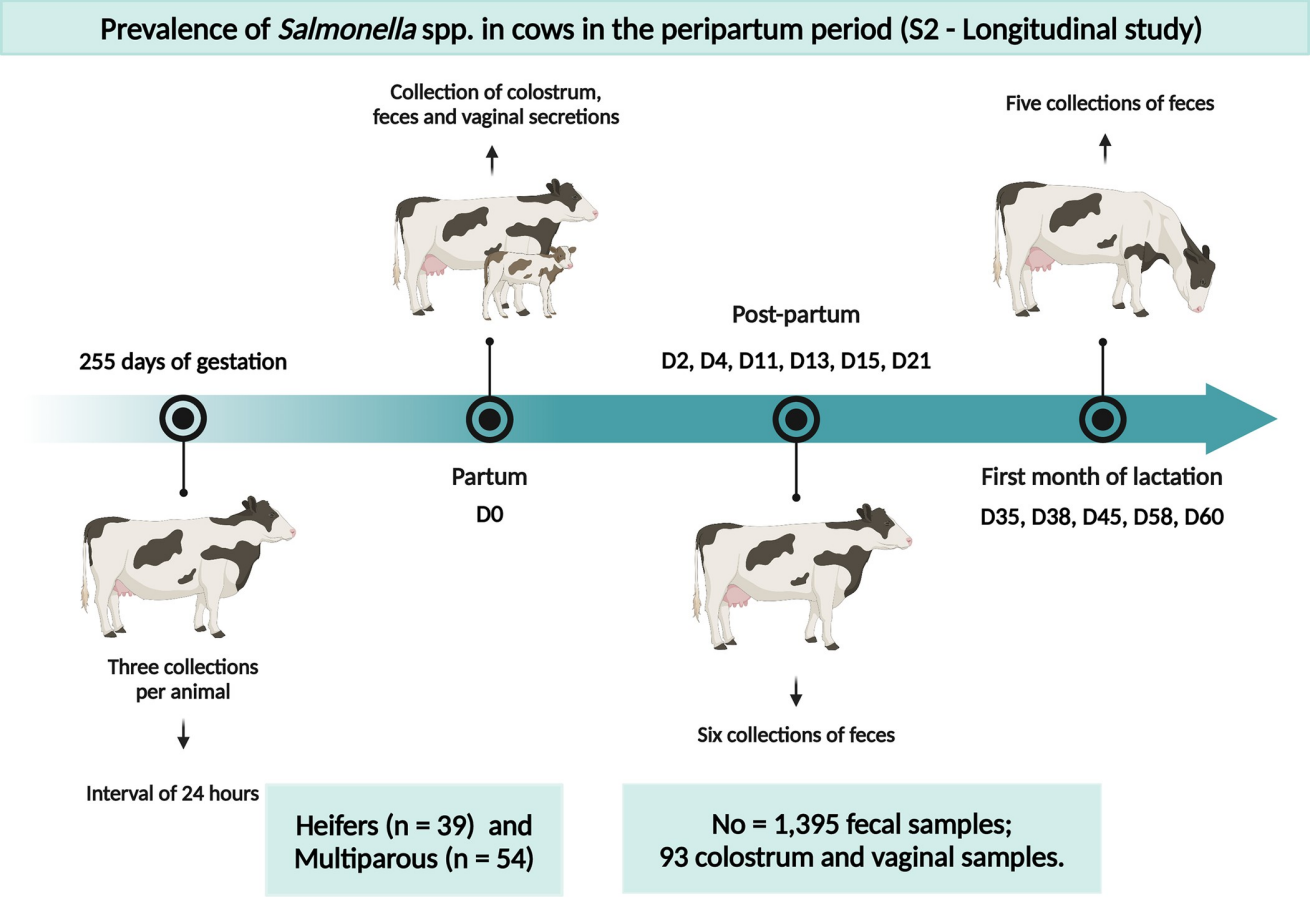
Graphical representation of the fecal sampling scheme in the longitudinal study (S2), focusing specifically on animals in the peripartum period.

After the first phase (S1), a longitudinal study was conducted, focusing on heifers and cows during the peripartum period. Only females without any evident clinical disease in the third trimester of gestation (approximately 255 days of gestation), according to the artificial insemination records on the farm, were included. A total of 39 heifers and 54 multiparous Holstein cows were monitored over 90 days. During this period, fecal samples were collected at 15 different time points, totaling 1,395 fecal samples (Fig 2). Colostrum and vaginal secretion samples were collected from all animals at the time of calving.

### Animals with symptoms suggestive of salmonellosis

Throughout the two experiments (S1 and S2), all animals that died on the farm with clinical signs suggestive of salmonellosis (n = 40) were subjected to postmortem examinations to investigate *Salmonella* spp. in the intestinal and biliary contents, cavity fluids (when ascites and hydrothorax were present), lungs, and lymph nodes.

### Isolation and characterization of *Salmonella* spp.

To minimize the impact of sample collection and processing time, all primary isolation steps were carried out in a laboratory setup on the farm itself. To detect *Salmonella* spp., fecal samples or swabs from extraintestinal organs were inoculated in Rappaport Vassiliadis broth (Merck, Germany), and incubated at 37°C. After 24 hours, the samples were plated on Hektoen Enteric Agar (Acumedia, USA), and incubated at 37°C. Following confirmation of the isolation, suspected strains were stored at -20°C in BHI broth supplemented with 20% glycerol, and sent to the Anaerobic Laboratory of the School of Veterinary Medicine at the Federal University of Minas Gerais for identity confirmation. The isolates were subjected to DNA extraction [20], followed by species-specific PCR to confirm the *Salmonella* genus [21]. Samples with confirmed identities were subjected to serotyping, according to the White-Kauffmann-Le Minor scheme [18].

### Antimicrobial susceptibility testing

The antimicrobial susceptibility of the isolates was tested using the agar disk diffusion method, as previously described by the Clinical and Laboratory Standards Institute [22]. In total, 15 antimicrobials were tested, representing 12 classes: amikacin (30 μg), amoxicillin/clavulanic acid (30 μg), ampicillin (10 μg), azithromycin (15 μg), cephalothin (30 μg), ciprofloxacin (5 μg), chloramphenicol (30 μg), colistin (10 μg), ceftriaxone (30 μg), fosfomycin (200 μg), gentamicin (10 μg), meropenem (30 μg), nitrofurantoin (300 μg), sulfamethoxazole/trimethoprim (25 μg), tetracycline (30 μg). *Escherichia coli* ATCC® 25922 was used as a control. *Salmonella* isolates resistant to three or more classes of antibiotics were considered multidrug-resistant. (MDR) [23].

### Whole genome sequencing and comparative genomics

Seven *Salmonella* Dublin strains (15%) were subjected to whole-genome sequencing. Two strains isolated from feces, one from colostrum, and four from necropsied animals (three from lungs and one from biliary contents) were selected at random (Table A in S1 File). The strains were incubated on Mueller–Hinton agar at 37°C for 24 h. Genomic DNA was extracted using the Wizard Genomic DNA Purification (Promega, USA). Genome sequencing was performed using the Illumina HiSeq platform (mid-out 2 × 150 bp cycles), while the raw data were analyzed using FastQC (Babraham Bioinformatics, Cambridge, England), retaining only paired reads with Phred quality of 30 or higher and a minimal size of 50 nucleotides.

The assembly was performed using SPAdes 3.5.0 in the careful mode [24]. GAP filling and polishing were performed using a Pilon [25]. ResFinder 4.1 [26–28] and PlasmidFinder 2.1 [29, 30] were applied to identify the determinants of acquired antimicrobial resistance and conjugative plasmid replicons, respectively. MLST 2.0 was used to determine sequencing types from these genome sequences [26, 27, 31].

The contigs were subjected to SNP analysis using CSIPhylogeny [32], with a minimal Z-score of 1.96 and a minimal depth at the SNP position of 10x. Strain 8814, isolated from bile content in the present study (accession number SAMN42384890), was used as a reference in the SNP analysis. Strains of *S*. Dublin isolated from animals and humans in Brazil between 2003 and 2016 were obtained from the Bacterial and Viral Bioinformatics Resource Center (BV-BRC) (https://www.bv-brc.org/) and previous studies [12] (Table B in S1 File). All trees were generated using iTOL online, with a midpoint rooting [33].

### Statistical analysis

Data analyses were performed using R software version 3.6.1 [34]. Differences in prevalence between groups were tested using logistic regression. Differences between groups were evaluated using multiple pairwise comparison tests (*pairwise*) with Tukey’s correction. The association between two categorical variables was assessed using the Fisher’s exact test.

## Results

### Prevalence of *Salmonella* spp. in the herd (S1)

**Fig 3:**
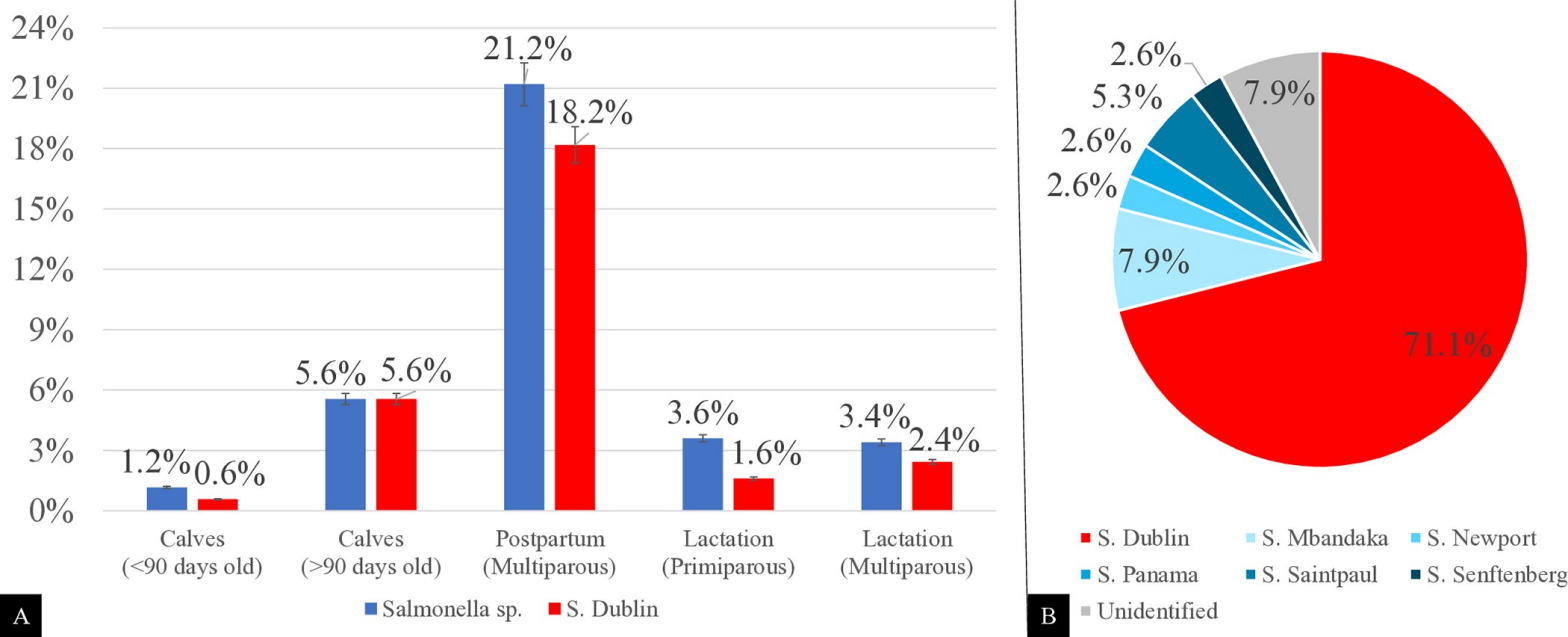
A—Distribution of *Salmonella* sp. and *S*. Dublin among the different categories of the studied herd. B—Distribution of the different serotypes of *Salmonella* among the categories of the studied herd.

To characterize and evaluate the prevalence of *Salmonella* spp. in the different categories of dairy herds, three fecal collections were performed from 759 animals with an interval of 48 hours, totaling 2,277 samples (S1). In this cohort, 38 samples from 26 cattle (3.4%) tested positive for *Salmonella* spp. (culture followed by PCR). Age-linked prevalence rates varying from 1.1% in animals up to 60 days of age to 21.2% in the postpartum phase of multiparous cows (Fig 3). Multiparous cows in the postpartum phase had 4.5 (95% CI; 1.2–16.7; p = 0.024) times higher odds of shedding *Salmonella* spp. compared to cows in the other life stages assessed.

Six serotypes were identified among the 38 *Salmonella* spp. isolates, with *S*. Dublin representing more than two-thirds of isolates (Fig 3) (27/38, 71.1%). *S*. Dublin was found in three of the five categories evaluated.

### Elimination of *Salmonella* spp. in the peripartum period (S2)

**Fig 4:**
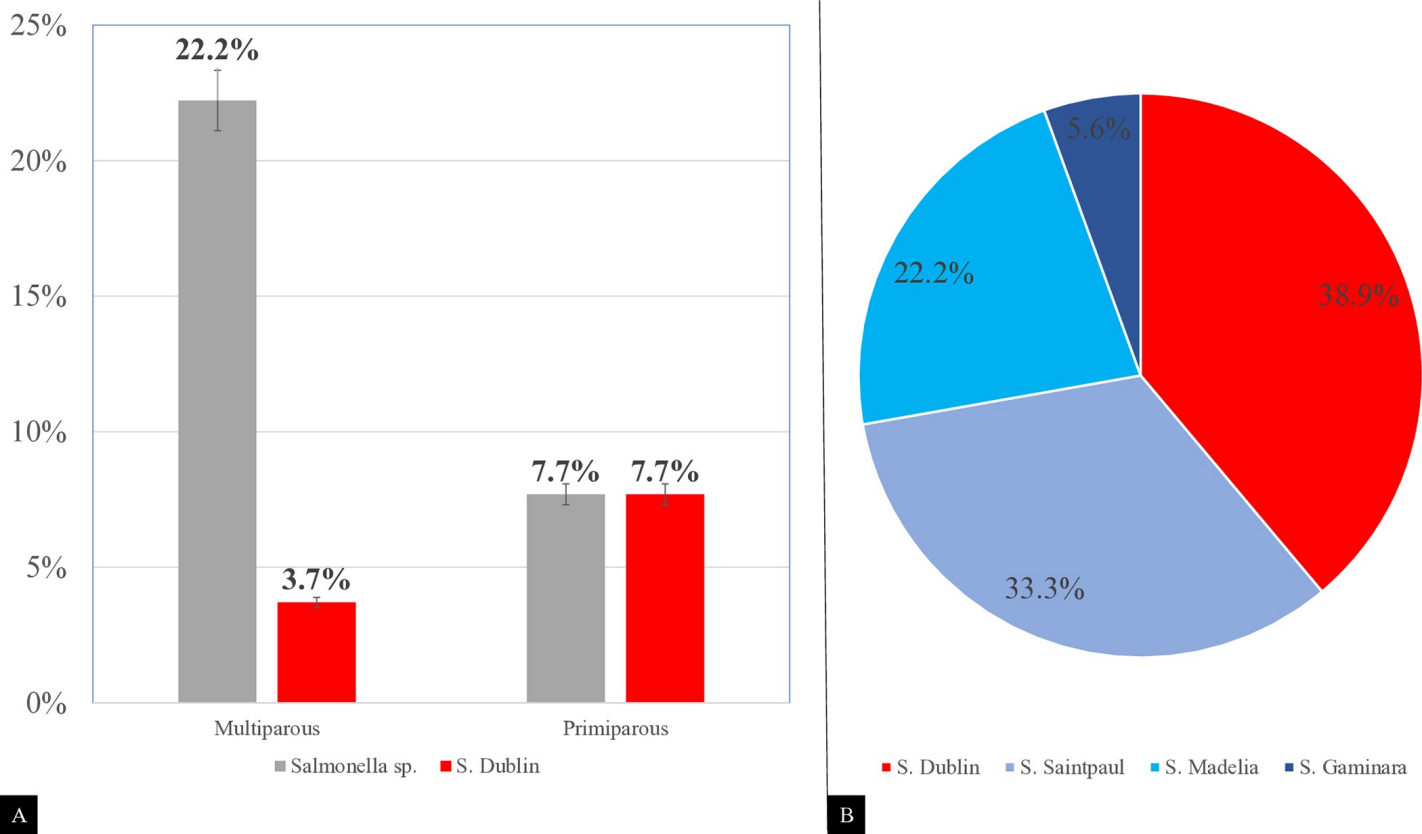
A—Prevalence of *Salmonella* spp. shedding during the peripartum period in multiparous and primiparous cows. B–Distribution of different *Salmonella* serotypes among the animals studied.

**Fig 5:**
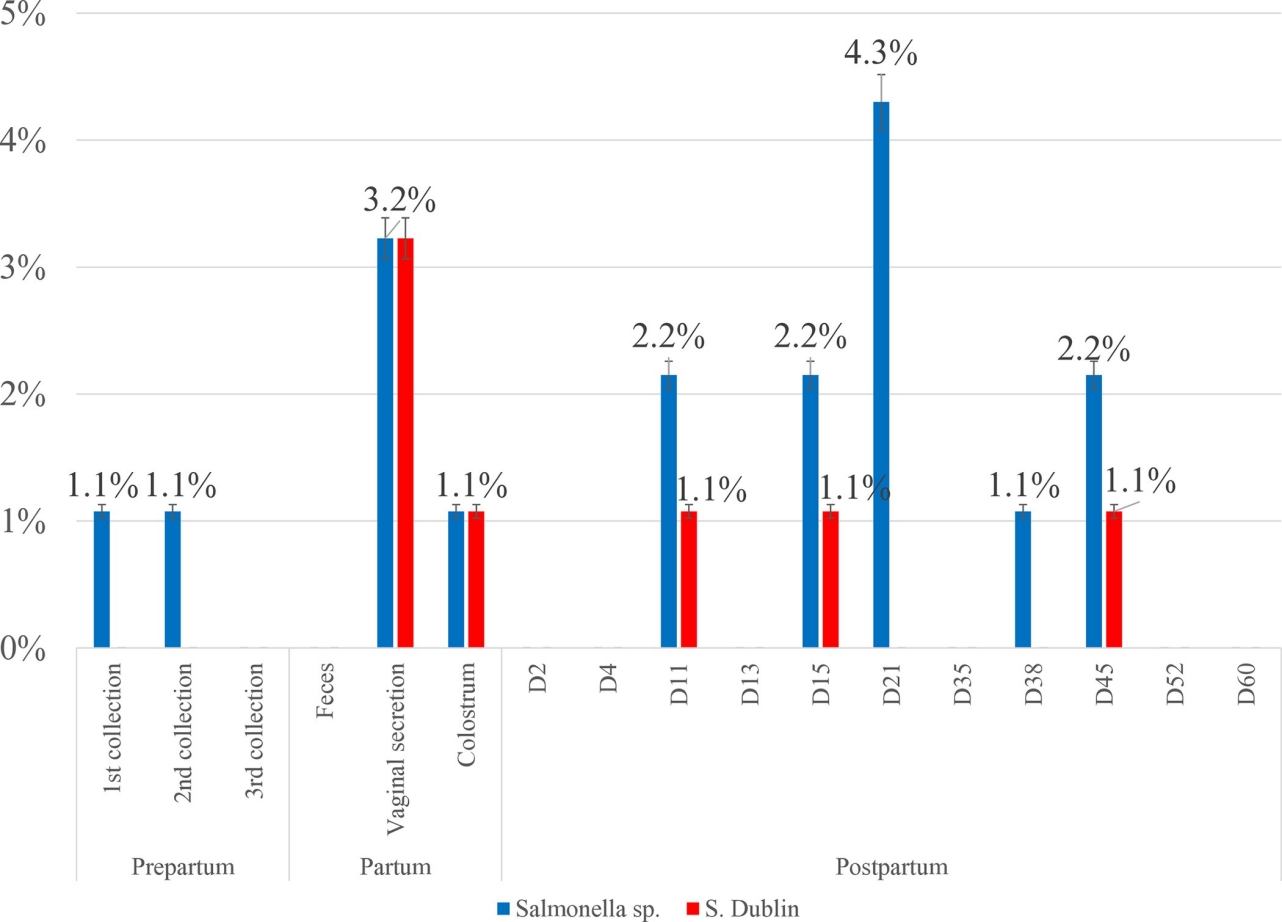
Prevalence of *S*. Dublin shedding and other serotypes during the prepartum, parturition, and postpartum periods.

Of the 1,395 fecal samples collected from animals during the pre- and postpartum periods (S2), *Salmonella* spp. were isolated from 18 samples collected from 15 animals (16.1%) (Fig 4). None of the animals tested positive for *S*. Dublin in prepartum collections. During parturition, *Salmonella* spp. were isolated from one (1.1%) colostrum sample and three (3.2%) vaginal secretion samples. At 60 days postpartum, *Salmonella* spp. were isolated between days 11 and 45 (Fig 5). Throughout the evaluation period, four serovars were identified, with *S*. Dublin and *S*. Saintpaul being the most frequently detected (38.9% and 33.3%, respectively) (Fig 4). Only *S*. Dublin was isolated from vaginal secretions and colostrum samples (Fig 5). There was no statistically significant difference in isolation between primiparous and multiparous animals.

### Clinical cases and post-mortem examination

**Fig 6:**
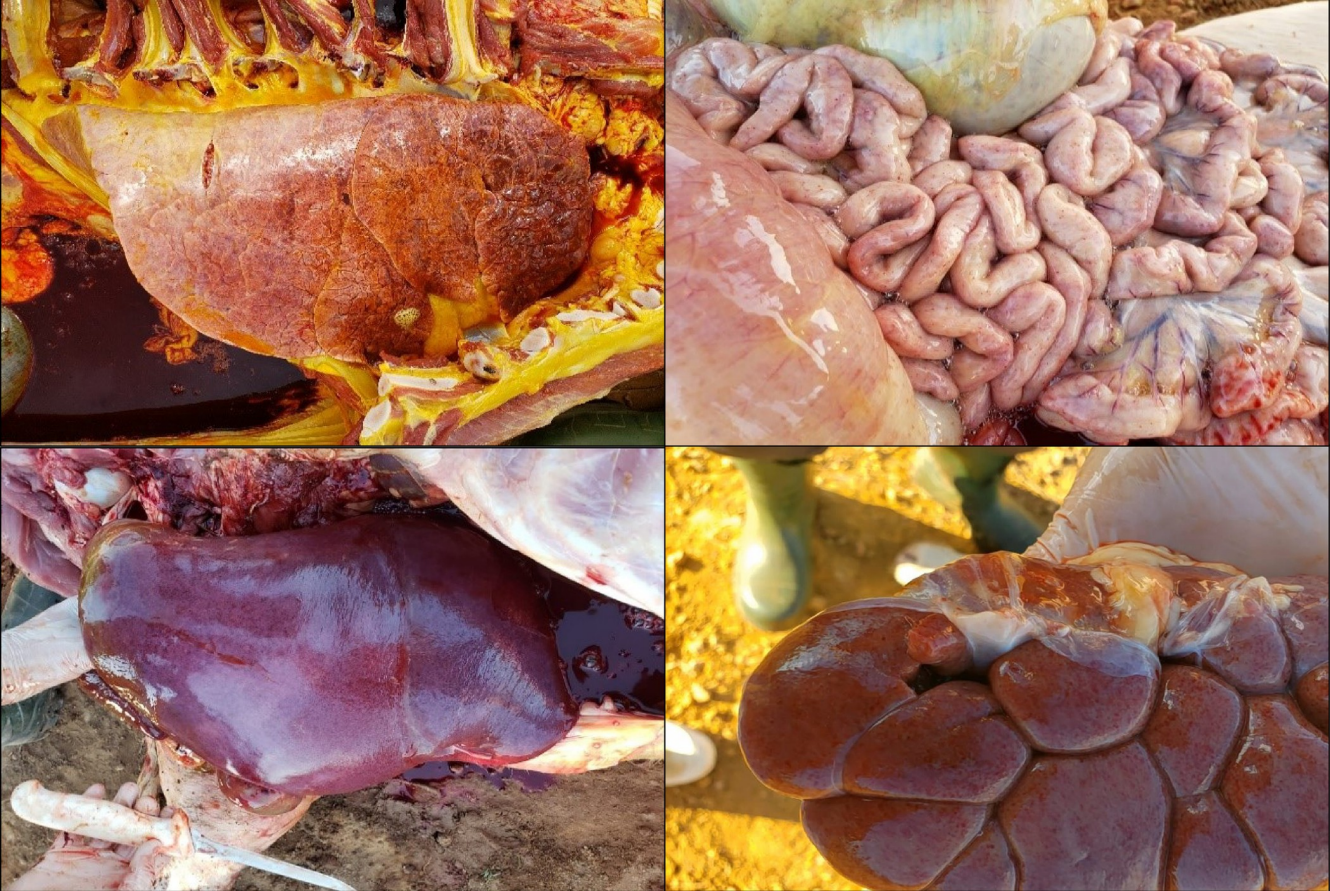
Necropsies of calves aged between 91 and 150 days in whom *Salmonella* sp. were isolated. A) Lung with severe edema, pronounced jaundice, and diffuse petechiae. B) Intestine with diffuse petechiae along the serosa, with areas of suffusion in the serosa. C) Enlarged liver with irregular coloring and rounded edges. D) Kidney with an irregular surface and diffuse petechiae throughout the surface.

Throughout both experiments (S1 and S2, totaling four months), 40 animals, all from the same category (aged between 91 and 150 days old), exhibited signs indicative of septicemic salmonellosis. The clinical presentation of these cows was characterized by jaundice, fever, and apathy. These animals were necropsied, revealing similar lesions in all individuals, including pulmonary congestion; petechiae on the surface of the intestines, kidneys, and bladder; hepatomegaly; and splenomegaly (Fig 6). Of the 40 necropsied animals, 39 (97.5%) tested positive for *S*. Dublin in the extraintestinal samples.

### Antimicrobial susceptibility

**Fig 7:**
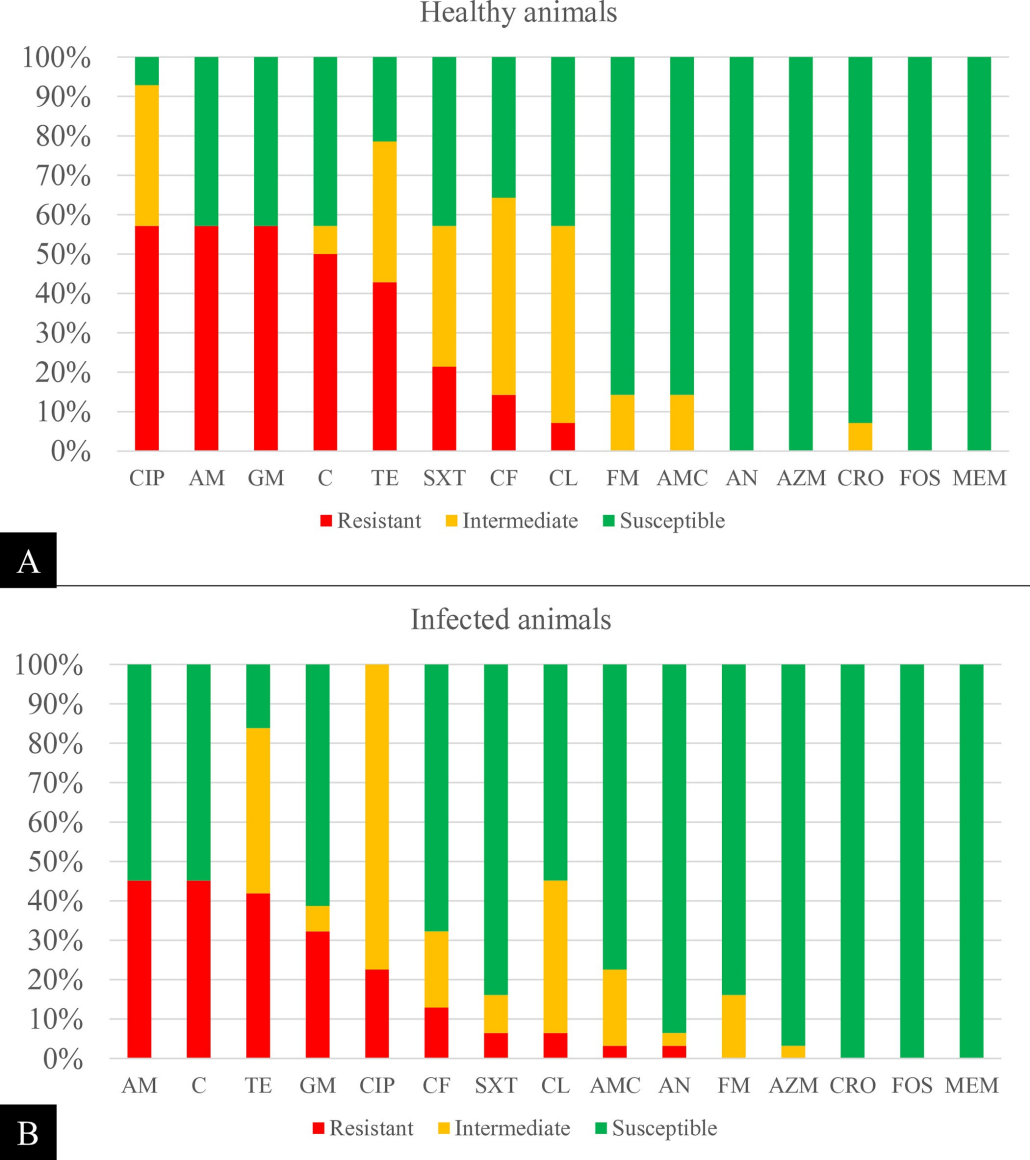
Antimicrobial resistance profile of *S*. Dublin samples isolated from healthy cattle (A, fecal shedding: n = 10; vaginal secretion: n = 3; colostrum n = 1) and from animals subjected to post-mortem examination (B, infected animals) on a dairy farm. AM = ampicillin/penicillins; C = chloramphenicol/phenicols; TE = tetracyclines; GM = gentamicin; CIP = ciprofloxacin/fluoroquinolone; CF = cephalosporins; SXT = sulfamethoxazole + trimethoprim; CL = colistin; AMC = amoxicillin + clavulanic acid; AN = amikacin; FM = nitrofurantoin; AZM = azithromycin; CRO = ceftriaxone; FOS = fosfomycin; MEM = meropenem.

A total of 45 *S*. Dublin isolates were subjected to antimicrobial susceptibility testing, of which 14 (31.1%) were from apparently healthy animals (isolated from feces (n = 10), colostrum (n = 1), or vaginal secretions (n = 3)) and 31 (68.9%) were extracted from the extraintestinal organs of animals with a confirmed case of salmonellosis (Fig 7). Most of the isolates (35/45, 77.8%) were resistant to at least one of the classes of antimicrobials tested, whereas 22 (48.9%) were classified as multidrug resistant (MDR). Approximately half of the isolates were resistant to penicillin (48.9%), followed by phenicols (46.7%), tetracyclines (42.2%), and aminoglycosides (40%). One-third (15/45, 33.3%) of the isolates were resistant to fluoroquinolones. Isolates resistant to first-generation cephalosporins or phenicols were associated with MDR profiles, whereas isolates from necropsy samples were associated with resistance to fluoroquinolones (p<0.05).

### Genomic characterization and comparative genomics

**Fig 8:**
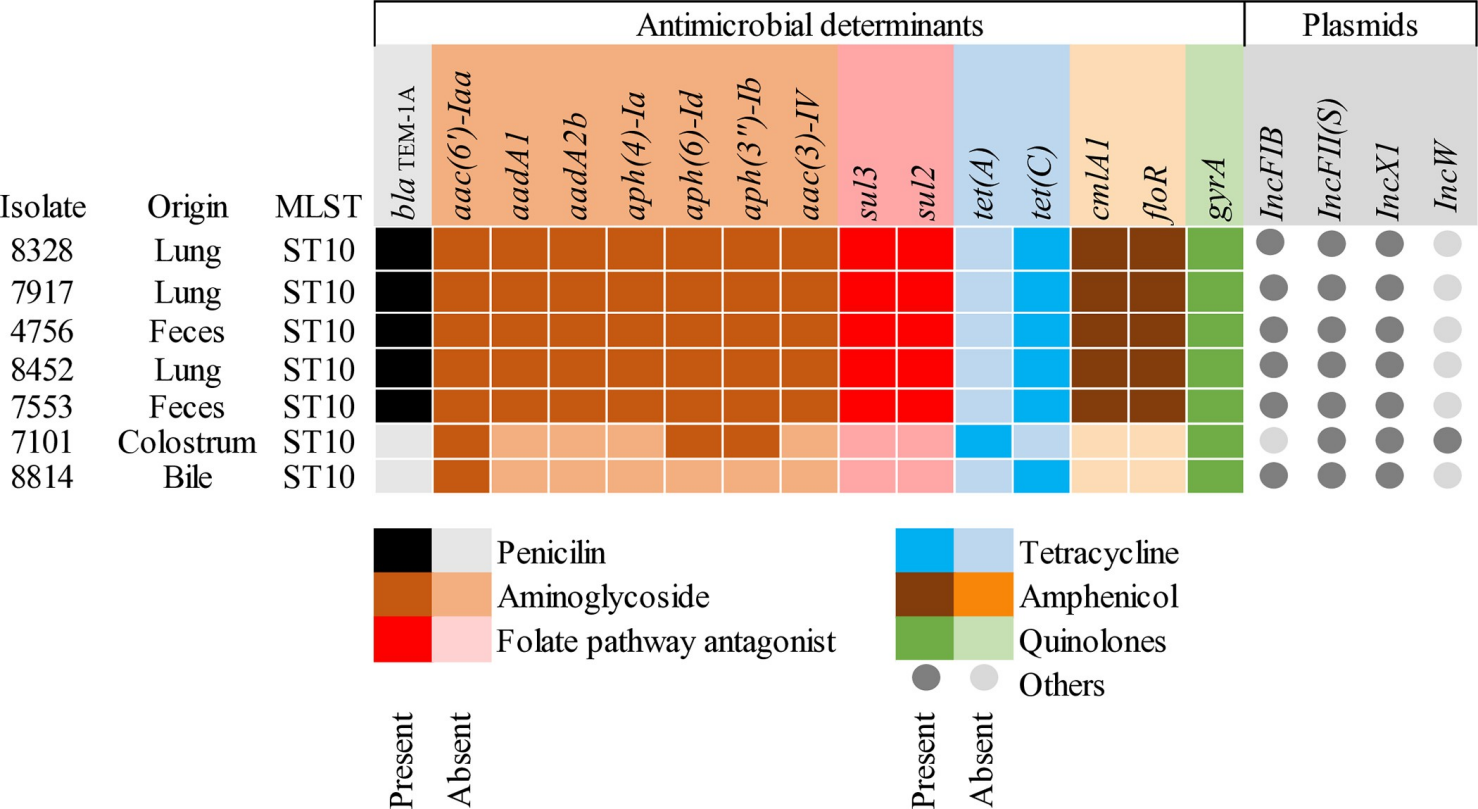
Resistance genes and plasmids found in the *S*. Dublin sequenced strains.

MLST analysis revealed that all strains sequenced in this study belonged to ST10, founder of the clonal complex 53 (CC53). All seven isolates showed resistance to aminoglycosides, tetracyclines, and fluoroquinolones, whereas five strains exhibited resistance to phenicols, penicillins, and folate antagonists (Fig 8). Four plasmids were identified from the sequenced samples. Two of them, IncFII(S) and IncX1, were identified in all samples, whereas IncFIB was present in six of the seven samples. The resistance determinants identified in each plasmid are listed in the Table C in S1 File.

The samples were compared with *S*. Dublin isolates from humans and animals obtained in previous studies. Among the 15 resistance genes identified in the isolates in this study, only four were identified in strains analyzed in prior studies in Brazil [12, 13]. The gene *aac(6’)-Iaa*, which confers resistance to aminoglycosides, the mutation in the *gyrA* gene, associated with fluoroquinolone resistance, and the genes *tet(A)* and *tet(C)*, associated with tetracycline resistance, were found in all isolates in the present study.

**Fig 9:**
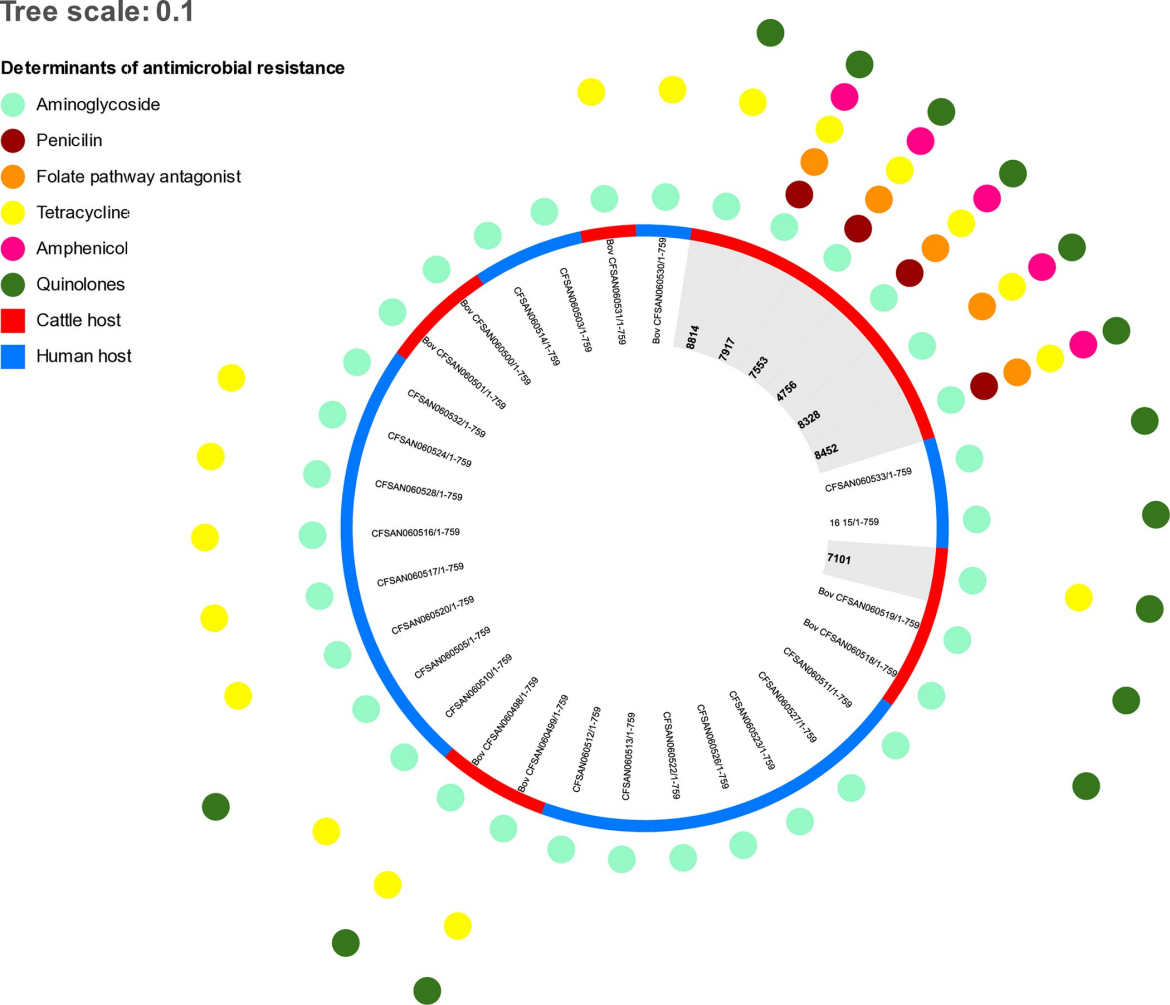
The phylogenetic tree of single nucleotide polymorphisms (SNPs) found in the genome of seven *S*. Dublin isolates obtained from healthy cattle or diagnosed with clinical salmonellosis (marked in gray). *S*. Dublin isolates from previous studies in Brazil [12] were included for comparison. The colored dots indicate the presence of resistance genes to different classes of antimicrobials, while the red and blue colors indicate the type of host of the isolate (bovine and human, respectively).

In the SNP analysis, high similarity was observed among the isolates in this study (Fig 9), with three strains from the lungs and two from feces showing between seven and 13 SNPs. Further, a comparison of the isolates from this study with strains from previous studies indicated little variation between *S*. Dublin isolates from animals and humans. cgMLST analysis further revealed that the samples in this study had between zero and three allele differences among themselves, except for the colostrum sample, where the difference ranged between 44 and 46 alleles. In relation to strains from other studies used for comparison, the isolates from this study showed between one and 77.

## Discussion

### Fecal shedding of *S*. Dublin in adult animals

In dairy production systems, salmonellosis leads to economic losses in various forms, ranging from reduced productivity to abortion, enteric and respiratory disorders, and outbreaks of high lethality [5, 6, 35]. In the present study, cases of salmonellosis were markedly concentrated in calves aged 90 to 150 days, in contrast to most reports in the literature that primarily described cases in calves up to 30 days of age, or in adult cows [5, 36–39]. This difference in the affected category and the large number of affected animals prompted us to conduct a study aimed at elucidating the main animal categories responsible for pathogen elimination on farms and antimicrobial resistance of the isolates.

In the present study, cows in the postpartum phase stood out because of the high prevalence of *Salmonella* spp. in feces (21.2%) (Fig 4), representing at least four times the likelihood of isolation compared to the other categories studied. The prevalence of *Salmonella* spp. varied from 0.6% to 5.6% in other categories. Interestingly, 86% of the isolates from multiparous cows belonged to the Dublin serovar, which is highly adapted to the bovine host [5, 19]. Some animals can become chronic carriers, shedding microorganisms into the environment primarily through feces, milk, and colostrum, facilitating the continuous colonization of susceptible animals that may develop clinical signs [5–7].

To better understand the dynamics of *S*. Dublin shedding in cows during the postpartum period, a longitudinal study (S2) was conducted with animals from 30 d prepartum to 60 d postpartum (totaling a period of 90 d). *S*. Dublin was obtained at parturition from colostrum and vaginal secretion samples, but only on day 11 postpartum in fecal samples (Fig 5). These results indicate that animals in the postpartum period, especially at the time of parturition and up to 45 days postpartum, exerted the greatest pressure of *Salmonella* spp. dissemination in the studied herd. Several hypotheses have been proposed to explain the increased shedding of *Salmonella* Dublin during this period. One suggests that the metabolic changes known to occur in periparturient dairy cattle may influence the shedding of various pathogens, including *Salmonella* sp., however the exact mechanisms involver remain unclear [40, 41]. Additionally, some researchers have suggested that this phase is associated with alterations in the microbiota, which may trigger the onset of *Salmonella* shedding; however, this hypothesis has not yet been confirmed [42]. Nevertheless, the present study highlights the role of healthy adult animals, particularly during the postpartum period, as a potential source of infection for calves [2, 3].

In addition to fecal shedding, *S*. Dublin was also isolated from one colostrum sample in the present study, supporting the results of previous studies indicating both colostrum and milk as potential sources of contamination in susceptible animals [1, 43, 44]. Isolation was further performed on the vaginal secretions of an animal in the immediate postpartum period, suggesting another possible source of infection for calves during parturition. *S*. Dublin may be present in the uterus of asymptomatic carrier animals, enabling the birth of infected calves or abortions [4, 45, 46].

Except for animals in the postpartum period, the prevalence of *Salmonella* spp. in lactating cows was low (between 3.4% and 3.6%), even lower than that reported in other studies, which reported a prevalence ranging from 9.1% to 92% [47–50]. Various factors may have contributed to the significant variation observed among these studies, primarily sampling techniques, culture media, seasonality, farm management, herd size, and animal nutrition [48, 51, 52]. Similar to other categories, the most positive isolates obtained in this category belonged to the Dublin serovar, contributing to the maintenance of the agent in the herd [52]. The authors of prior studies noted that the lactation process is a stress factor that could influence the shedding of pathogens, such as *Salmonella* sp. [40, 41], posing a risk of infection for lactating animals, as well as for individuals involved in milk production [47, 51, 53].

### Fecal shedding and infection by *S*. Dublin in calves

In nursing calves (<90 days old), the prevalence (1.2%) was also lower than that reported in most similar studies, which detected *Salmonella* in up to 11.3% of this age group [19, 54–56]. Several hypotheses may explain the reduced prevalence. The use of discarded milk with antibiotic residues, a common practice on the studied properties, has also been reported to influence the isolation rate of *Salmonella* spp. in previous studies [38, 55, 57]. Furthermore, some calves under 90 days of age are commonly administered antimicrobials to treat diarrhea and bovine respiratory diseases (fluoroquinolones, penicillins, tetracyclines, and chloramphenicol), which may have negatively influenced the isolation rate in this category, and could partially explain the resistance profile of the isolates obtained in the present study.

Approximately half of the *Salmonella* sp. isolates obtained from nursing calves were from the Dublin serovar. Interestingly, *S*. Dublin is highly pathogenic to young calves, with numerous reports of cases and outbreaks in animals up to 30 days old [58–60]. However, in the present study, there was no history of salmonellosis in this age group, while no animals in this category showed any signs of the disease throughout the four months of the study, in contrast to previous works [38, 58].

Calves between 91 and 150 days of age represent a category that merited particular attention in the present study. The prevalence of *Salmonella* spp. in the feces of calves in this category (5.5%) was lower than that reported in a similar study of calves in Denmark [19]. On the other hand, several cases of lethal septicemic salmonellosis were confirmed in this age group during the study. This finding contrasts with those of previous studies, indicating a higher incidence of the disease in animals under 30 days old [37, 38, 58]. One hypothesis to explain the strong occurrence of salmonellosis in this animal category is the presence of parasitic diseases such as anaplasmosis and babesiosis (commonly caused by *Babesia bigemina* and *B*. *bovis*). Indeed, the occurrence of salmonellosis has commonly been reported in association with other conditions in various species, such as infections with *Plasmodium falciparum* and *Schistosoma mansoni* in humans and canine distemper in wildlife, among others [61–66]. In cattle, bovine parasitic sadness has been suggested to be an important risk factor for *S*. Dublin infections by other authors [67]. Other factors may also be involved in the high occurrence of salmonellosis in this age group, such as a decline in passive immunity typical of this age group, weaning stress, and changes in diet and environment, including moving from individual housing to group pastures [68–70].

In addition to *S*. Dublin, other serotypes of *Salmonella* spp. were found in the present study, albeit in much lower proportions, ranging from 2.9% to 7.9%. Nevertheless, it is important to point out that these serotypes are not negligible, as they are recognized to be significant in cattle, and are even associated with cases of salmonellosis in humans, such as Newport [71, 72], Panama [73, 74], Mbandaka [75, 76] and Infantis [77, 78].

### Antimicrobial resistance and resistance determinants

One-third of the *S*. Dublin isolates in the present study were resistant to fluoroquinolones, which is higher than the proportions reported in other studies [47, 79]. Genomic sequencing suggested that the S83F mutation in *gyrA* was the main determinant of resistance. Notably, fluoroquinolones are of great significance for treating severe cases of salmonellosis in humans [5, 80, 81], making *Salmonella* spp. resistant to this class a top priority for the development of new antimicrobials [82]. One hypothesis to explain the high frequency of resistance to fluoroquinolones is the extensive use of enrofloxacin in various farm protocols, such as diarrhea, anaplasmosis, and pneumonia in calves. Studies have suggested that the increased detection of *Salmonella* sp. strains with *gyrA* gene mutations responsible for this resistance appears to be strongly driven by the high use of fluoroquinolones in livestock [83–87].

Almost half of the isolates in this study exhibited resistance to penicillins (46.9%), phenicols (42.9%), and tetracyclines (42.9%), which is similar to the rates reported in other studies on *Salmonella* spp. in cattle [1, 80, 88]. Both the *florR* and *cmlA1* genes, which are responsible for resistance to phenicols, and the *bla*_TEM-1A_ gene, which is associated with resistance to beta-lactams, were found in a significant portion of the isolates subjected to genomic sequencing, corroborating the phenotypic profile observed. Interestingly, samples resistant to phenicols or cephalosporins were associated with multidrug resistance, which has already been described in previous works [89, 90]. This association is likely related to the acquisition of mobile genetic elements by these isolates, which can simultaneously carry resistance genes against various classes of antimicrobials [83]. In this context, it is worth noting the detection of plasmids IncFII(S) and IncX1 in the majority of the isolates, which corroborates the findings of other studies [12, 91–93]. Both plasmids are associated with the emergence of multidrug-resistant strains responsible for causing severe infections, including in humans [94].

### Genomic characterization and comparative genomic

MLST analysis revealed that all the strains in this study belonged to ST10, which is commonly associated with the serovar Dublin [12, 92, 95, 96]. cgMLST and SNP analyses confirmed that the isolates found in the feces of healthy animals are extremely similar to those found in the necropsy of calves with salmonellosis, indicating that the same agent circulating among the carrier animals is responsible for the clinical cases and deaths on the farm [97, 98]. Simultaneously, the similarity between the isolates of this study and those from previous outbreaks in cattle in Brazil corroborates the already known high clonality of the *S*. Dublin genome, and reinforces the importance of adopting biosecurity measures on dairy farms to prevent the spread of the agent among herds in Brazil [91, 93, 99, 100]. This study also revealed a high similarity between some isolates from the studied farm and strains causing illnesses in humans in Brazil, similar to a recent study [12]. It is important to emphasize that *S*. Dublin infection in humans is commonly associated with high rates of hospitalization and mortality worldwide, including in Brazil [12, 96], and that recent studies have indicated an increase in the occurrence and severity of the disease in humans. Collectively, these results reinforce the idea of an epidemiological link between bovine and human salmonellosis, indicating the need for a One Health approach for better control and prevention of this disease.

## Conclusion

The present study suggests that adult animals in the postpartum period are a significant source of *S*. Dublin on dairy farms, whereas calves up to 150 days of age were the most affected by the infection. The detection of multidrug-resistant isolates, including those resistant to fluoroquinolones, and the high similarity between strains from infected humans highlights the importance of this study and the need for biosecurity measures in herds to reduce transmission, as well as a One Health approach for better control and prevention of this disease in a broader context.

## Supporting information

S1 File Supplementary tables A to C. (DOCX)

S2 File SNP matrix. (TXT)

S3 File Rawdata. (XLSX)
