# Supplementary material for: Dynamics of Salmonella Dublin infection and antimicrobial resistance in a dairy herd endemic to salmonellosis
Source: PLoS One. 2025 Jan 23;20(1):e0318007. doi: 10.1371/journal.pone.0318007 (PMC11756764; doi:10.1371/journal.pone.0318007)
Supplement: S1 File — (DOCX) [file pone.0318007.s001.docx]

**Table A: Sequence type, origin of isolate, and accession number of the *Salmonella* Dublin strains isolated from bovines in on a dairy farm in Brazil.**

| Isolate | Site of infection | MLST | Genome Accession |
| --- | --- | --- | --- |
|  |  |  |  |
| 4756 | Feces | ST10 | SAMN42384884 |
| 7101 | Colostrum | ST10 | SAMN42384885 |
| 7553 | Feces | ST10 | SAMN42384886 |
| 7917 | Lung | ST10 | SAMN42384887 |
| 8328 | Lung | ST10 | SAMN42384888 |
| 8452 | Lung | ST10 | SAMN42384889 |
| 8814 | Bile | ST10 | SAMN42384890 |

MLST: Multilocus sequence type (Achtman Scheme); ST: sequence type.

**Table B: Isolation host, year and accession number of the *Salmonella* Dublin strains used for comparison with the strains of the present study.**

| **Strain** | **Host** | **Year** | **GenBank Accessions** |
| --- | --- | --- | --- |
| CFSAN060498 | Cattle | 2003 | QBTA00000000 |
| CFSAN060499 | Cattle | 2003 | QBTB00000000 |
| CFSAN060500 | Cattle | 2003 | QBTC00000000 |
| CFSAN060501 | Cattle | 2003 | QBTD00000000 |
| CFSAN060503 | Human | 2003 | QBTF00000000 |
| CFSAN060505 | Human | 2003 | QBTH00000000 |
| CFSAN060510 | Human | 2005 | QBTM00000000 |
| CFSAN060511 | Human | 2005 | QBTN00000000 |
| CFSAN060512 | Human | 2005 | QBTO00000000 |
| CFSAN060513 | Human | 2005 | QBTP00000000 |
| CFSAN060514 | Human | 2005 | QBTQ00000000 |
| CFSAN060516 | Human | 2007 | QBTS00000000 |
| CFSAN060517 | Human | 2010 | QBTT00000000 |
| CFSAN060518 | Cattle | 2013 | QBTU00000000 |
| CFSAN060519 | Cattle | 2013 | QBTV00000000 |
| CFSAN060520 | Human | 2013 | QBPE00000000 |
| CFSAN060522 | Human | 2013 | QBPC00000000 |
| CFSAN060523 | Human | 2013 | QBPB00000000 |
| CFSAN060524 | Human | 2013 | QBPA00000000 |
| CFSAN060526 | Human | 2014 | QBOZ00000000 |
| CFSAN060527 | Human | 2014 | QBOY00000000 |
| CFSAN060528 | Human | 2014 | QBOX00000000 |
| 16_15 | Human | 2015 | RZIU00000000 |
| CFSAN060530 | Cattle | 2015 | QBOV00000000 |
| CFSAN060531 | Cattle | 2015 | QBOU00000000 |
| CFSAN060532 | Human | 2015 | QBOT00000000 |
| CFSAN060533 | Human | 2016 | QBOS00000000 |

**Table C – Resistant determinants detected in the plasmids in each *Salmonella* Dublin isolate included in the present study**

|  | IncFIB | IncFII(S) | IncX1 | IncW | Not detected in the plasmids |
| --- | --- | --- | --- | --- | --- |
| 8328 | *aadA1; aadA2b; cmlA1; sul;* | + | + | - | *aph(4)-Ia; aph(6)-Id; aph(3'')-Ib; aac(3)-IV; blaTEM-1a; floR; sul2* |
| 8814 | + | + | + | - |  |
| 7101 | - | + | + | *aph(6)-Id; aph(3'')-Ib; tet(A)* |  |
| 7553 | *aadA1; aadA2b; cmlA1; sul;* | + | + | - | *aph(4)-Ia; aph(6)-Id; aph(3'')-Ib; aac(3)-IV; blaTEM-1a; floR; sul2* |
| 4756 | *aadA1; aadA2b; cmlA1; sul;* | + | + | - | *aph(4)-Ia; aph(6)-Id; aph(3'')-Ib; aac(3)-IV; blaTEM-1a; floR; sul2* |
| 8452 | *aadA1; aadA2b; cmlA1; sul;* | + | + | - | *aph(4)-Ia; aph(6)-Id; aph(3'')-Ib; aac(3)-IV; blaTEM-1a; floR; sul2* |
| 7917 | *aadA1; aadA2b; cmlA1; sul;* | + | + | - | *aph(4)-Ia; aph(6)-Id; aph(3'')-Ib; aac(3)-IV; blaTEM-1a; floR; sul2* |
